# Internal Migration as a Social Determinant of Occupational Health and WASH Access in Myanmar

**DOI:** 10.5334/aogh.3381

**Published:** 2021-11-11

**Authors:** Heidi West, Marlar Than, Thinzar Win, Khin Thein Oo, Kyi Khaing, Thin Thin Aye, San Myint Yi, Su Yi Myo, Su Yi Toe, Maja Milkowska-Shibata, Kristin Ringstad, Can Meng, Tomoyuki Shibata

**Affiliations:** 1 University of California Los Angeles, US; 2 Meiktila University of Economics, MM; 3 Mandalay University, MM; 4 Meiktila University, MM; 5 Mandalar Degree College, MM; 6 Yadanabon University, MM; 7 Mandalay City Development Committee, MM; 8 University of Medicine, Mandalay, MM; 9 Global Environmental Health LAB, US; 10 Yale University, US; 11 Northern Illinois University, US

## Abstract

**Background:**

Migration is at an all-time high worldwide, and despite increased focus on international migrants, there is little evidence about internal migrants’ exposures to socioeconomic, occupational, and environmental risk factors in low-and middle-income countries.

**Objective:**

The aim of this study was to examine differences in occupational health and access to water, sanitation, and hygiene (WASH) between internal migrants and non-migrants.

**Methods:**

A face-to-face survey (n = 937) was conducted in Mandalay, Myanmar. Bivariate and multivariate analysis included traditional social determinants such as education, income, occupation, gender, age, and location in addition to internal migration status.

**Findings:**

The majority of internal migrants (23% of the total sample) were labor migrants (67.3%), and while common social determinants (e.g., household income, education, and gender) were not statistically different between migrants and non-migrants, these groups reported different occupational profiles (p < 0.001). Migrants had higher odds of being street vendors (AOR = 2.26; 95% CI 1.33–3.85; p = 0.003) and were less likely to work labor jobs such as in factories or construction (AOR = 0.44; 95% CI 0.19–1.00; p = 0.051) when controlling for age, gender, education, and location. Internal migrants had significantly greater probabilities of experiencing some injuries and illness symptoms, such as cuts, vomiting, coughing, heatstroke, and diarrhea at work (p < 0.001). Compared to non-migrants, migrants’ households were approximately three times more likely (AOR = 3.45; 95% CI 2.17–5.62; p < 0.001) to have an unimproved source of drinking water and twice as likely (AOR = 1.98; 95% CI 1.10–3.58; p < 0.05) to have unimproved sanitation facilities in their homes.

**Conclusions:**

The results underscore the importance of considering internal migration as an aspect of social determinants analyses, and the need for targeting appropriate WASH interventions to address inequities.

## 1. Introduction

Migration is at an all-time high worldwide, and despite the increased focus on international migrants and the known vulnerabilities associated with migration status [1,2,3,4], less is known about the particulars of internal migrants, and the links between their migration experience and health [5]. Internal migration has been shown to affect maternal health services utilization [6,7,8], child mortality [9], physical and mental health [10,11,12]. Migration and health literature highlight the positive and negative impacts of migrating parents on psychosocial and physical health outcomes for children [13], as well as the positive effects children’s migration can have on elders’ health [14]. Relationships between migration and family outcomes are well documented. However, specific outcomes related to the environmental and occupational risk factors facing internal migrants and their families are still understudied.

Migration and work are inextricably linked. Most of the world’s migrants are labor migrants and even as migrant workers make up a growing percentage of the workforce, domestic labor markets are becoming increasingly segmented with migrants concentrated in certain sectors or occupations [15,16]. Internal migrants face unique vulnerabilities that can increase occupational illness and injury. These vulnerabilities persist even when controlling for more traditional social determinants such as occupation, gender, age, income, and education [17,18]. Occupational risks, such as exposure to unhealthy air or lack of access to clean water and sanitation on the job, are of particular concern as they relate to some of the leading preventable causes of morbidity and mortality and are situated within the broader development context of the Southeast Asia region [19,20,21].

The 2030 Agenda for Sustainable Development and its 17 Sustainable Development Goals (SDGs) include a focus on occupational protections (SDG 8 Decent Work and Economic Growth), but also prioritize water, sanitation, and hygiene (WASH) [22]. Access to clean water and sanitation (SDG 6) at work and at home is critical for preventing waterborne diseases such as diarrhea. Almost two-million deaths, 3.3% of global deaths and 13% of under-five deaths, as well as 4.5% of global disability-adjusted life-years (DALYs) could have been prevented with adequate WASH [23]. WASH related illnesses are linked to social determinants such as education, which influences health and hygiene behavior [24,25,26]. Not only is equity at the center of addressing WASH with a social determinants lens [27], but policy and resource allocation decisions rely on scientific identification of target populations for interventions to reduce death and disability. This study contributes to understanding the intersections of social determinants and environmental and occupational health through the lens of migration in a historically understudied location.

Myanmar, a lower-middle-income country, has experienced social, political, and economic transition over the last decade, accompanied by extensive internal migration. Approximately 20% of Myanmar’s population is internal migrants (9.39 million people), the vast majority moving for economic or family related reasons [28]. The International Labour Organization (ILO) has emphasized the vulnerable status of internal migrants in Myanmar while advocating for improved governance of labor migration [18]. There is little evidence about inequities faced by internal migrants in Myanmar and especially in relation to their exposure to socioeconomic, occupational, and environmental risk factors. While there is growing attention to the connections between health, development and socio-economic factors [29], including migration, there are limited studies comparing health indicators across migrant groups within Myanmar and work to date has focused on maternal and reproductive health, child health, and infectious diseases with a concentration of studies in the Myanmar-Thailand border region [7,30,31,32]. A focus on migration, occupation, and WASH in Myanmar is warranted considering both the vast and growing numbers of internal labor migrants, unequal access to the benefits of recent development initiatives, and the burden of disease. For example, diarrhea and acute respiratory infections still account for almost a quarter of all under-5 deaths [33].

There is growing use of social determinants analyses to understand the impact of factors such as education, housing, and employment on health [29] and to identify target populations and appropriate entry points for health interventions [34]. While there is growing attention to the connections between health, development, and socioeconomic factors, including migration [4,35], there are limited studies comparing health indicators across migrant groups in Myanmar. The objective of this study was to examine migration as a social determinant of occupational health and access to WASH, when controlling for conventional social determinants (e.g., education, occupation, income, age, gender, township, and health status).

## 2. Methods

### 2.1 Study Site

Mandalay City, the second largest city in Myanmar (population 1.2 million), is the economic center of upper Myanmar and its location on the Ayerwady River promotes trade, commerce, and travel. As such, Mandalay is a prime location for studying internal migration. Two townships in the Mandalay District, Chanayethazan and Amarapura, were purposively selected for their different characteristics. Chanayethazan Township, classified as urban, covers the downtown area of the city and is a mix of commercial and residential neighborhoods. Amarapura Township, classified as a rural area, is located about 13 kilometers from Chanayethazan (approximately 45 minutes by car) on Taungthaman Lake and is comprised of large areas of farmland and the textile industry.

### 2.2 Data Collection

This cross-sectional study employed a survey tool that was developed based on existing studies in the region [24], WHO guidelines [36], and the indicator framework of the SDGs [22]. The questionnaire consisted of migration status questions and eight other sections on demographics, housing characteristics, water and food access, sanitation and hygiene, general health, occupational health, maternal health, and child health. The questionnaire was validated by local faculty members as to the question choice, comprehensibility, and validity. The survey was further pilot tested on a small sample to validate its accuracy, cultural appropriateness, and burden on the participants. The questionnaire was developed in English and translated into Burmese, the official language in Myanmar.

Data collection took place as part of the second and third “Workshop and Collaborative Research for the Sustainable Development Goals (SDGs)” program in Myanmar approved by the Myanmar Ministry of Education and directed by Global Environmental Health LAB in 2016 and 2017. The Institutional Review Board at Northern Illinois University, DeKalb, Illinois, USA approved the study protocol (IRB # HS16-0174: Community and occupational health associated with the SDGs) prior to data collection. In person interviews with respondents 18 years of age or older who provided informed consent were conducted in Burmese by trained faculty members from Yadanabon University. Interviews lasted approximately 45–60 minutes and were conducted between June 2016 and September 2017. Participant selection was done using the convenience sampling method combined with a geographic sampling plan developed by the Department of Geography at Yadanabon University. Women were oversampled as data were collected as part of a larger study focused on women with children under five, yielding a predominantly female sample (77%) for this analysis.

### 2.3 Measures

Migration status was the main predictor variable and was determined using the following definition provided by the International Organization for Migration: “a person who moves away from his or her place of usual residence, whether within a country or across an international border, temporarily or permanently, and for a variety of reasons” [37]. Internal migration in this study included change of usual residence between townships, districts, states, and/or regions, consistent with definitions used by the International Organization for Migration and the Myanmar Department of Population, Ministry of Immigration and Population in census enumeration. Migration status was divided into two categories – migrants and non-migrants and one subcategory of migrants, forced migrants. Migration status was self-reported and not limited to moves occurring within a particular time frame. Outcomes were all dichotomous and fell into two categories: a) Occupational illnesses and injuries (four specific illness symptoms, five specific injuries, plus “other”) and b) WASH (improved source of drinking water, improved source of water for other household use, improved sanitation facility, handwashing behavior).

Covariates included age (continuous), gender (binary), education (individual and household; categorical and binary), income (continuous and binary), occupation (categorical and binary), type of housing (permanent or temporary, building structure/materials; categorical and binary), township (rural/urban; binary), and self-reported health status (categorical and binary). The main income variables were binary measures indicating whether a household fell below the survey respondents’ median income or the poverty line. The household poverty level was based on SDG Target 1.1: Eradicate extreme poverty, with extreme poverty defined as $1.25 or less per person per day [22]. In many settings such as Myanmar, other measures of poverty such as dwelling characteristics, assets and access to safe water and sanitation can often be more informative, especially when used in combination with income measures [38,39]. Accordingly, we included a measure of dwelling characteristics that captured the building type/materials and level of permanence (e.g. apartment, bungalow/brick house, temporary hut). Education was measured both on the individual and also the household levels. The education measures were operationalized to include categorical and binary variables with cutoffs for primary and nine years of compulsory education (up to lower secondary or middle school). These different specifications of the education variables were tested across the different outcomes and in both bivariate and multivariate analyses. These analyses also included checks for collinearity and correlations with variables such as occupation and income. Occupational categories were derived from the Myanmar census and consultation with labor experts in the Department of Economics at Yadanabon University. Categories with zero responses were excluded, and categories with fewer than 2% of overall respondents (~fewer than 20 respondents) were reviewed for possible combinations with similar occupational categories. This resulted in the combining of government occupations and categorizing the currently “unemployed” into “other” because their prior occupations varied and were not captured by this survey. The final categorical variable included eight occupation categories (***Table 2***). In addition to the categorical version of the occupation variable, we tested dummy variables for each occupation group.

### 2.4 Statistical analyses

Data collected from the face-to-face interviews were entered into Microsoft Excel 2010 as the 2016–2017 Mandalay Community and Occupational Health (MCOH) dataset (n = 937) [40]. Analyses were conducted using Stata 15 software (StataCorp. 2017). Chi-square, t-tests, and Fisher’s exact tests were used to compare descriptive outcomes between internal migrants and non-migrants and by occupation category. We analyzed the effects of internal migration on health risks and behaviors, and considered the effects of labor segmentation by location and migration status. Logistic regression models were fit to determine the associations between migration status and dichotomous outcomes related to occupational health and WASH while controlling for factors such as income, gender, education, township, and health status.

## 3. Results

### 3.1 Study Population

Twenty-three percent of respondents (n = 220) were internal migrants, approximately 4% higher than the national statistics [28], but not unexpected given the location near the second largest city in Myanmar. The rural township, Amarapura, was home to about 60% of the non-migrants, while the urban township, Chanayethazan, a major downtown hub, had almost 60% of the migrants (p < 0.001). Migrants were not significantly different than non-migrants in terms of social and demographic measures including ethnicity (97.7% Bamar), religion (98.4% Buddhist), marital status (73% married), and mean age (42.8 ± 14.1 years old).

There were no statistical differences between migrants and non-migrants across conventional socio-economic measures such as education and household income (***Table 1***). The majority of households (63.6%) had at least one person who had completed high school (11 years) or university education and over 92% of all respondents had completed at least primary education. However, 40% of respondents had not completed compulsory education (secondary school, 9 years). Median household income was 250,000 Myanmar Kyats per month (approximately $200 USD). A large proportion of the study respondents (35.6%) lived in extreme poverty, defined as $1.25 or less per person per day [22]. On a secondary measure of poverty, housing type, there were significant differences between internal migrants and non-migrants with 31.5% of migrants living in temporary (1–3 years) huts or bamboo houses, while only 20.7% of non-migrants lived in such units (23.2% overall).

**Table 1:** Socio-demographic characteristics of internal migrants and non-migrants in the Mandalay District.


VARIABLE	TOTAL (N = 937) NO. (%)	NON-MIGRANTS (N = 717) NO. (%)	MIGRANTS (N = 220) NO. (%)	*P*

Urban – Chanayethazan	416 (44.4)	288 (40.2)	128 (58.2%)	<0.001

Average age	42.8 ± 14.1	43.2 ± 14.2	41.5 ± 13.7	0.113

Female	715 (77.0)	549 (77.1)	166 (76.9)	0.938

Self-reported health				

Poor/Fair (ref: good/excellent)	327 (35.8)	244 (35.1)	83 (38.1)	0.418

Participant’s education^a^				0.106

None and non-standard curriculum	66 (7.1)	46 (6.5)	20 (9.1)	–

Primary school	307 (33.0)	242 (34.0)	65 (29.5)	–

Secondary school	253 (27.2)	199 (28.0)	54 (24.5)	–

High school	165 (17.7)	115 (16.2)	50 (22.7)	–

University	140 (15.0)	109 (15.3)	31 (14.1)	–

Household highest education^a^				0.174

None and non-standard curriculum	29 (3.1)	18 (2.5)	11 (5.0)	–

Primary school	115 (12.4)	89 (12.6)	26 (11.9)	–

Secondary school	193 (20.8)	142 (20.1)	51 (23.3)	–

High school	277 (29.9)	209 (29.6)	68 (31.1)	–

University	312 (33.7)	249 (35.2)	63 (28.8)	–

Household in extreme poverty^b^	263 (35.6)	205 (34.9)	58 (38.2)	0.458

Lives in temporary hut/bamboo house (ref: all other housing types)	215 (23.2)	146 (20.7)	69 (31.5)	0.001


^a^ p value shown for categorical variable not individual dummy variables. ^b^ Equal or less than 1.25 USD/person/day.

### 3.2 Occupation and Health

The majority of internal migrants moved for employment/economic related reasons (67.3%). This percentage was higher than the 34.3% nationwide average for employment only induced migration, but not inconsistent with rates in urban settings; in Mandalay 51.5% of migrant men and 25.7% of migrant women are labor migrants [28]. Occupational segmentation by migration status and location were both observed, but stratified analysis by location and migration status showed that internal migration status had a more consistent and stronger associations with occupation group than urban versus rural location. For example, in stratified bivariate analysis by urban/rural location, migration status was a significant predictor of occupation group for both rural and urban residents (p < 0.01 urban, p < 0.001 rural), but when stratified by migration status, location was only a significant predictor of occupation group for non-migrants (p < 0.001). Additionally, in stratified multivariate logistic regression models for specific occupations controlling for age, gender, and education, living in an urban area was a significant predictor of the probability of being a street vendor for non-migrants (p = 0.006), but not for internal migrants.

It was notable that while income and education levels were statistically indistinguishable between migrant and non-migrant respondents, their occupational profiles were different. Internal migrants were more likely to be street vendors and drivers, rather than working in government, agriculture, or labor related occupations (p < 0.001) (***Table 2***). Not a single internal migrant in this sample worked in the construction industry and only seven worked in factories. Approximately 62% of construction workers resided in the rural township, while most street vendors and drivers were located in the urban township. When controlling for education, gender, age and rural vs. urban location, occupation and migration status were still significantly associated. For example, compared with non-migrants internal migrants were approximately two times as likely to be street vendors (AOR = 2.26; 95% CI 1.33–3.85; p = 0.003) and significantly less likely to be government workers (AOR = 0.30; 95% CI 0.11–0.79; p = 0.015). Migrants and non-migrants were equally likely to be business owners (AOR = 0.97; 95% CI 0.65–1.43; p = 0.866).

**Table 2:** Occupational health of internal migrants and non-migrants.


ILLNESS/INJURY	OCCUPATION	NON-MIGRANT	MIGRANT	OCCUPATION	NON-MIGRANT	MIGRANT

	Construction/Factory (n = 60)	53 (7.9)	7 (3.4)*	Own Business (n = 196)	154 (22.8)	42 (20.3)

Injury (any)		5 (9.6)	0 (0.0)		10 (7.3)	2 (5.7)

Cuts		4 (7.7)	0 (0.0)		5 (3.6)	2 (5.7)

Illness (any)		6 (11.8)	2 (28.6)		12 (9.0)	14 (38.9)***

Heatstroke		4 (8.0)	1 (14.3)		11 (8.2)	9 (25.7)**

Vomiting		1 (2.1)	0 (0.0)		0 (0.0)	3 (10.3)**

Diarrhea		1 (2.1)	0 (0.0)		0 (0.0)	2 (6.9)*

Coughing		1 (2.1)	1 (16.7)		3 (2.3)	9 (27.3)***

	Driver (n = 23)	11 (1.6)	12 (5.8)***	Street Vendor (n = 68)	40 (5.9)	28 (29.0)***

Injury (any)		0 (0.0)	3 (27.3)		1 (3.2)	10 (47.6)***

Cuts		0 (0.0)	0 (0.0)		1 (3.2)	10 (47.6)***

Illness (any)		2 (50.0)	3 (42.9)		5 (17.2)	13 (50.0)*

Heatstroke		2 (50.0)	3 (42.9)		2 (6.9)	12 (46.2)***

Vomiting		0 (0.0)	0 (0.0)		0 (0.0)	0 (0.0)

Diarrhea		0 (0.0)	0 (0.0)		0 (0.0)	0 (0.0)

Coughing		2 (50.0)	0 (0.0)		3 (10.7)	9 (45.0)*

	Farmer (n = 53)	49 (7.3)	4 (1.9)**	Housewife (n = 229)	169 (29.0)	60 (29.0)

Injury (any)		1 (3.8)	1 (25.0)		2 (9.5)	5 (26.3)

Cuts		1 (4.0)	0 (0.0)		2 (9.5)	4 (21.1)

Illness (any)		4 (14.8)	0 (0.0)		3 (17.6)	4 (23.5)

Heatstroke		4 (14.8)	0 (0.0)		3 (17.6)	2 (11.8)

Vomiting		1 (3.8)	0 (0.0)		0 (0.0)	1 (5.9)

Diarrhea		1 (3.8)	0 (0.0)		0 (0.0)	0 (0.0)

Coughing		4 (14.8)	0 (0.0)		0 (0.0)	0 (0.0)

	Government (n = 42)	37 (5.5)	5 (2.4)	Other (n = 211)	162 (24.0)	49 (23.7)

Injury (any)		2 (6.7)	2 (40.0)		18 (17.3)	9 (24.3)

Cuts		2 (6.7)	2 (40.0)		9 (9.1)	2 (5.6)

Illness (any)		8 (26.7)	2 (40.0)		23 (23.0)	12 (30.8)

Heatstroke		8 (26.7)	2 (40.0)		13 (13.1)	8 (21.6)

Vomiting		0 (0.0)	0 (0.0)		4 (4.3)	4 (11.4)

Diarrhea		3 (10.7)	0 (0.0)		3 (3.2)	5 (14.3)*

Coughing		3 (10.7)	0 (0.0)		14 (14.9)	6 (17.6)

	Overall (n = 882)	675 (76.5)	207 (23.5)***			

Injury (any)		40 (9.6)	32 (22.5)***			

Cuts		24 (5.8)	20 (14.7)***			

Illness (any)		67 (16.5)	50 (35.0)***			

Heatstroke		48 (11.9)	37 (26.4)***			

Vomiting		6 (1.6)	8 (7.0)**			

Diarrhea		8 (2.1)	7 (6.1)*			

Coughing		33 (8.6)	25 (20.3)***			


Statistical significance based on chi-square and Fisher’s Exact tests between migrants and non-migrants. *** p < 0.001, ** p < 0.01, * p < 0.05 95% CI in parentheses.

Overall, internal migrants experienced significantly more occupational illnesses symptoms such as vomiting, coughing, heatstroke, and diarrhea compared with non-migrants. In bivariate analysis by occupation group and migration status, there were not a lot of differences in occupational health between migrants and non-migrants who were in the same jobs. However, for street vendors, the most common occupation for migrants in this study, and respondents who own their own businesses, migrants experienced certain injuries and illnesses at higher rates than their non-migrant colleagues in the same occupation (***Table 2***). When controlling for other risk factors, including occupation, gender, location, and income, migrants had more than twice the odds of non-migrants of developing heatstroke at work (AOR = 2.74; 95% CI 1.44–5.19, p < 0.01), and more than three times the odds of developing any illness symptoms at work (AOR = 3.2; 95% CI 1.81–5.07, p < 0.001). While migrants had significantly higher percentages of work injuries such as cuts in bivariate analysis, migration status was not a significant predictor of injuries in multivariate regressions, controlling for occupation, education, age, gender, and income status.

### 3.3 WASH

There were significant differences between migrants and non-migrants across a range of water, sanitation, and hygiene (WASH) measures (***Table 3***). Only 74.4% of migrants had access to improved drinking water, while 89.8% of non-migrants reported similar access. Differences were smaller, but still significant for improved household water (88.6% of non-migrants, 80.8% of migrants) and sanitation facilities (93.1% of non-migrants, 86.4% of migrants).

**Table 3:** WASH Access and behavior.


VARIABLE	TOTAL (N = 937) NO. (%)	NON-MIGRANTS(N = 717) NO. (%)	MIGRANTS (N = 220) NO. (%)	*P*

Lives in urban area	416 (44.4)	288 (40.2)	128 (58.2)	<0.001

Lives in temporary hut or bamboo house	215 (23.2)	146 (20.7)	69 (31.5)	0.001

Improved source of drinking water	796 (86.2)	636 (89.8)	160 (74.4)	<0.001

Improved source of household water	800 (86.8)	627 (88.6)	173 (80.8)	0.004

Improved sanitation facility	842 (91.5)	657 (93.1)	185 (86.4)	0.002

Always wash hands with soap^c^	342 (38.2)	272 (39.8)	70 (33.2)	0.085

After using toilet	807 (90.8)	629 (92.5)	178 (85.2)	0.001

Before hand or breastfeeding children	381 (76.8)	301 (80.3)	80 (66.1)	0.001

Before cooking or preparing food	622 (87.1%)	500 (89.1)	122 (79.7)	0.002


Statistical significance based on chi-square tests between migrants and non-migrants. ^c^ Handwashing: Each outcome variable is measured as 1 = always 0 = not always. “Always” variable is before/after each of 5 activities (toileting, cleaning child’s bottom, feeding, eating and cooking).

Living in a temporary hut or bamboo housing unit, living in the urban area, and being a migrant were significantly associated with decreased access to WASH (improved drinking water, household water and sanitation). Compared to non-migrants, migrants were approximately three times less likely to drink water from an improved source (AOR = 3.49; 95% CI 2.17–5.61; p < 0.001), almost two times less likely to use an improved source of water for other household use (AOR = 1.90; 95% CI 1.15–3.13; p < 0.05), and half as likely to have improved sanitation facilities in their homes (AOR = 1.98; 95% CI 1.10–3.58; p < 0.05) when controlling for poverty, type of housing, location (rural/urban), and education (***Table 4***).

**Table 4:** Multivariable analysis of WASH access.


VARIABLE	IMPROVED SOURCE OF DRINKING WATER	IMPROVED SOURCE OF HOUSEHOLD WATER	IMPROVED SANITATION FACILITY

Non-Migrant	3.49***	1.90*	1.98*

	(2.17–5.61)	(1.15–3.14)	(1.10- 3.58)

Lives in a rural area	1.58	2.42***	1.19

	(0.981–2.55)	(1.47–3.97)	(0.641–2.19)

Lives in permanent housing	1.94*	3.48***	4.19***

	(1.15–3.25)	(2.07–5.85)	(2.39–7.37)

Compulsory Edu (HH)	1.06	1.14	3.30***

	(0.588–1.92)	(0.625–2.09)	(1.85–5.89)

Household above poverty line	1.78*	1.11	0.935

	(1.12–2.82)	(0.686–1.78)	(0.528–1.66)

Observations	713	716	717


Logistic regression (Odds Ratios). 95% Confidence Intervals in parentheses. Compulsory education or greater is at least 9 years for one household member. Poverty = $1.25 per person per day. *** p < 0.001, ** p < 0.01, * p < 0.05.

Internal migrants, in general, were approximately two times (AOR = 1.81; 95% CI 1.19–2.74; p < 0.001) less likely to always wash their hands with soap compared to non-migrants (***Table 5***). Specifically, migrants were approximately three times less likely to always wash their hands with soap after using the toilet (AOR = 2.80; 95% CI 1.66–4.72; p < 0.001) and before feeding children by hand or breastfeeding (AOR = 2.84; 95% CI 1.68–4.79; p < 0.001), and half as likely to wash with soap before cooking (AOR = 2.08; 95% CI 1.20–3.61; p < 0.001) when controlling for age, gender, education, income, and rural versus urban setting.

**Table 5:** Multivariable analysis of handwashing behavior: Wash hands with soap and water.


VARIABLE	ALWAYS – 5 ACTIVITIES	AFTER USING THE TOILET	BEFORE HAND OR BREASTFEEDING CHILDREN	BEFORE COOKING

Non-Migrant	1.81**	2.80***	2.84***	2.08**

	(1.19–2.74)	(1.66–4.72)	(1.68–4.79)	(1.20–3.60)

Age	0.984*	1.01	1.02	1.06***

	(0.972–0.998)	(0.993–1.03)	(0.997–1.04)	(1.04–1.09)

Female	2.10**	1.11	1.36	0.595

	(1.36–3.25)	(0.593–2.08)	(0.701–2.63)	(0.255–1.39)

Lives in rural area	1.04	1.09	0.899	1.06

	(0.737–1.48)	(0.637–1.87)	(0.530–1.52)	(0.614–1.85)

Median income +	0.809	1.33	1.01	0.699

	(0.587–1.12)	(0.804–2.187)	(0.631–1.63)	(0.424–1.153)

Compulsory Edu (Ind)	1.21	1.96*	0.817	1.53

	(0.855–1.71)	(1.16–3.33)	(0.480–1.39)	(0.882–2.66)

Observations	699	694	382	562


Logistic regression (Odds Ratios). *** p < 0.001, ** p < 0.01, * p < 0.05 95% CI in parentheses. All variables except age (continuous) are dichotomous. Median income = $200 USD per month. Compulsory education or greater is at least 9 years for the individual respondent. Each outcome variable is measured as 1 = always 0 = not always. “Always” variable is before/after each of 5 activities (toileting, cleaning child’s bottom, feeding, eating and cooking).

## 4. Discussion

Migrants and non-migrants in this study were ethnically, linguistically, and religiously homogenous, and are not statistically different in terms of education and income, yet migrants still face occupational and environmental health risks at much higher rates than their local counterparts. Internal migrants in this study experience labor force segmentation, a higher burden of occupational related illness, and are more likely to live in temporary housing with inadequate access to improved WASH. These are diverse issues that span typical disciplinary divisions; however, their common thread is the persistent association with internal migration. Even when controlling for standard determinants such as age, gender, location, education, income, and occupation, migration poses its own risks separate from those associated with most social determinants. Internal migrants may face environmental and occupational health risks as a result of their vulnerability as migrants and separate from the effects of poverty or educational disparities. These risks are problematic in their own right in terms of health, well-being, and equity. However, they also have implications for the health outcomes of migrants and their families.

This study has several limitations including the lack of geographic, ethnic, and linguistic diversity of the sample. Statistics on nationwide migration patterns and other population level data were obtained through the 2014 Myanmar Population and Housing Census. This government census does not include data on approximately 1.2 million individuals who are not enumerated based on ethnic group, including over 1 million Rohingya and over 100,00 individuals in the Kachin and Kayin States. While this limits generalizability across diverse groups, the homogeneity of the sample across language, ethnicity and religion helps to control for these confounding factors that can influence both migration status and the outcomes of interest. The focus on the majority groups allows to better isolate the role of migration in the absence of other persistent vulnerabilities such as religious or ethnic based discrimination. As random sampling was not feasible, the study faces external validity threats associated with sampling bias as well as internal validity selection bias threats. Our sample has an overrepresentation of women and likely self-selection of respondents who had time to answer a lengthy survey meaning those in more demanding occupations may not represented. We cannot fully address bias in our estimates due to unobserved variables, but we are able to reduce the impacts through the use of large sample, inclusion of a broad range of observed covariates, and we conducted tests for correlations and associations across all variables in our analyses as part of model selection. Migration related questions only collected information on the primary respondent, and our analyses did not include duration since migration. This lack of household migration measures limits our ability to paint a complete picture of migration’s impact on household level health risks. Finally, the cross-sectional nature of the design prevents us from making causal inferences and further research is needed to understand the nuance and directionality of the associations presented herein. Regardless of these limitations, this study fills an important gap by providing evidence on the relationships between internal migration and health in Myanmar’s second largest city, an area with comparatively few scientific studies.

There are three key implications of this study for future research and policy considerations. First, migration should be included in analyses of social determinants of health. The type and measures of migration are often context specific. However, many data sets and censuses include basic migration data, and these should not be overlooked in favor of traditional social determinants. In analyzing health risks, the policies and programs that influence those risks, and/or the subsequent health outcomes, it is important to examine migrant communities in order to gain a more thorough picture of the relationships between migration and health [4]. Attention to the social and economic frameworks within which interventions are operating is essential to the study of the social determinants of health in analyzing outcomes, inequities, or disadvantages. Migrants and non-migrants, especially labor migrants in Myanmar, may not be statistically different in terms of income, yet other migration related factors may have negative impacts on health access and well-being including the limitations of temporary housing, experiences such as the trauma of forced migration, and distance from family and support centers. This study directs attention to occupational segregation and differential risk within occupation groups by migration experiences. An approach that can flesh out these differences can provide a more robust picture of public health and the need for systems to adapt with migration trends [17]. In order to support effective services for migrants, including targeted health interventions, we must have a clear picture of the needs, and also the strengths, of different migrant groups. Our study points to interesting socio-economic similarities between economic migrants and the general population. Analyses of social determinants of health need to look beyond income poverty in order to explain differences between and within countries [41]. This study highlights the need for further research to differentiate migrant groups within and between contexts. Additionally, this study has established a foundation upon which further research can be conducted to examine how the changes in social determinants attributable to migration link to other important domains such as healthcare access, well-being, and development. Analyzing migration with a social determinants lens allows for greater understanding of the complexities across migration groups and of the pushes and pulls of migration.

Second, migration related data collection and analyses disaggregated by migration status contribute to the agenda for global development and are in line with increased calls for attention to the complex relationships between migration and health. Our analysis contributes to global efforts for social change, such as the United Nations Sustainable Development Goals (SDGs) and local and international measurement of progress toward their achievement by 2030 [22]. The SDGs are the first time that a global development agenda has recognized the contributions of migration, and as a cross-cutting issue, migration is included in targets and indicators of ten out of the seventeen goals. There are specific targets for reducing occupational illness and injury and improving access to WASH, but looking at these rates disaggregated by migration status helps to ensure progress toward health equity. An important aspect of the SDGs is the applicability across all countries, high, middle, and-low-income, and the requirement to look beyond aggregate measures and averages to understand disparities within countries. The SDGs framework of this study can be applied in other contexts, and is particularly relevant in Asia where broad disparities exist within and between countries and in a region that sustains several of the world’s largest internal and international migration corridors [42]. This study further demonstrates the importance of including migration status questions in community health epidemiological studies. Doing so can facilitate more comprehensive social determinants analyses, and from a global perspective, help demonstrate differential migration effects in different settings through international comparisons. Studies such as ours help answer growing calls for attention to migration, including those in the newly adopted UN Global Compact for Migration. They are an important step toward understanding not just where individual communities stand in relation to global targets, but through what mechanisms progress is being made or hindered.

Finally, the inclusion of migration in health studies can provide evidence that assists policy makers in setting priorities, policy implementation, and resource allocation. Migration can provide a lens to evaluate the effectiveness of local, national, and international development projects [43], and highlight policy implications related to economic push and pull factors [44]. Structural inequities that impact health can be addressed through policy interventions and resource allocation. Migration is related to structural inequities. When seeking to identify health disparities, vulnerable population characteristics, and entry points for interventions, analyzing variables across migration groups is a valuable approach. For example, through identifying groups that are more vulnerable to WASH related illness, local governments and organizations in Myanmar can target water and sewer infrastructure projects. Lack of access to improved water and sanitation can make it very difficult to follow recommended handwashing practices [45]. As migrants had less access to improved WASH, it is not surprising that when it came to hygiene behavior, migration status also played a significant role in whether or not individuals followed recommended hand washing practices. The direct and indirect effects of internal migration on health and well-being can span families and generations. Future studies should expand on this work to explore linkages between the WASH and occupational health risk factors identified herein and health outcomes, particularly for vulnerable populations such as children and pregnant women. When seeking environmental and policy interventions to improve the health of communities, it is critical to understand these macro level determinants which may be a few steps removed from the long-term outcomes.

In shifting the focus from individual and proximate factors to social, structural, and macro level causes, what Link and Phelan coin as fundamental causes [46], this study not only has important implications for migration and health but offers evidence that supports inclusion of migration in social determinants tools and frameworks. In addressing layered vulnerabilities brought on by migration, labor market segmentation and housing inequities, policy makers can expand outreach strategies to include stakeholders in migrant communities and occupational representatives [47]. We find ourselves in times where migrant remittances outpace formal foreign aid and the international community has turned its sights on migrants. Internal and international migration has afforded many families the opportunity for better jobs, education, and poverty alleviation. However, vulnerabilities and inequities persist. The nuances of the relationships between migration, development and population health warrant attention both between and within national contexts.
